# Effects of Salt and Fat Combinations on Taste Preference and Perception

**DOI:** 10.1093/chemse/bjv079

**Published:** 2015-12-26

**Authors:** Dieuwerke P. Bolhuis, Lisa P. Newman, Russell S.J. Keast

**Affiliations:** Centre for Advanced Sensory Science, School of Exercise and Nutrition Sciences, Deakin University, 221 Burwood Highway, Burwood, Victoria 3125, Australia

**Keywords:** fat, fat taste sensitivity, salt, taste intensity, taste preferences

## Abstract

Fat and salt are a common and attractive combination in food and overconsumption of either is associated with negative health outcomes. The major aim was to investigate contributions and interactions of salt and fat on taste pleasantness and perception. The minor aim was to investigate individual fat taste sensitivity (detection threshold of oleic acid [C18:1]) on pleasantness for fat. In a complete factorial design, 49 participants (18–54 years, 12 males) tasted tomato soups with 4 different fat concentrations (0–20%) and 5 different salt concentrations (0.04–2.0%). The preferred concentration and the discrimination ability for both fat and salt were determined by ranking tests. Results show that salt and fat affected pleasantness separately (*P* < 0.01), with salt having the strongest effect. Fat concentrations 0%, 5%, and 10% did not differ in pleasantness, whereas 20% was less pleasant (*P* < 0.05). There were no interactions for fat and salt on pleasantness or saltiness and fattiness intensity. Fat taste sensitive participants preferred lower fat concentrations than less sensitive participants (*P* = 0.008). In conclusion, the strong effect of salt on pleasantness in this study suggests that salt, rather than fat, play a major role in the attraction to savory fatty foods.

## Introduction

Fat and salt both stimulate appetite (Blundell and Macdiarmid 1997; Leshem 2009) and are therefore an attractive combination in food. Many processed foods high in dietary fat also have high salt contents, like meat, cheese, crisps, fries, and many other savory foods. Mixtures of fat and salt are commonly found in high-fat, high energy dense snacks and fast foods. Overconsumption of both dietary fat and salt are associated with various negative health outcomes, for example, cardiovascular disease (Bray and Popkin 1998; Bray et al. 2004; Brown et al. 2009; He and MacGregor 2010).

There is increasing evidence that fatty acids (i.e., the breakdown products of dietary fat) are detected by the gustatory system and “fat” is considered as a sixth taste primary (e.g., Mattes 2009a; DiPatrizio 2014; Tucker et al. 2014; Keast and Costanzo 2015). The ability to taste and discriminate between fat concentrations varies between individuals and has been found to be inversely related to body weight (Stewart et al. 2010, 2011a, 2011b; Martínez-Ruiz et al. 2014), however, the area remain contentious as others have not found this relationship (Mattes 2009b, 2011). Previous research suggests that diet is an important factor influencing fat taste sensitivity and excessive consumption of dietary fat over 4 weeks attenuates fat taste sensitivity in lean subjects, while reducing dietary fat increases sensitivity in both lean and obese (Stewart and Keast 2012b).

It is important to unravel factors that contribute to the overconsumption of dietary fat. Salty/savory foods are major sources of dietary fat intake. Preference for foods high in both fat and salt are associated with high daily energy intakes and obesity (Cox et al. 1999; Méjean et al. 2014). Salt is added to a wide range of processed foods to increase the palatability (Booth et al. 1983; Dotsch et al. 2009), which consequently increases energy intake (Bouhlal et al. 2011; Bolhuis et al. 2012). Salt in food is highly palatable, however, the role of fat on palatability is less clear (Cox et al. 2015). We propose that salt plays an essential role in the palatability of savory fatty foods, indicating that salt concentrations drive preferences rather than fat concentrations.

Fat may influence the perception of saltiness in a food. Ohta et al. (1979) found that saltiness intensity was reduced in oily compared with aqueous media. Similarly, 2 other studies found that saltiness intensity increased when water increases in the emulsion, or reduced when oil increases in the emulsion (Rietberg et al. 2012; Suzuki et al. 2014). A proposed hypotheses was that oil may act as a barrier between salt and the salt taste channels. However, others found no effect of oil content on saltiness perception (Metcalf and Vickers 2002), or even an increased saltiness perception with higher oil concentrations in oil in water emulsions (Yamamoto and Nakabayashi 1999; Thurgood and Martini 2010).

The primary aim is to investigate independent contributions and interactions of salt and fat concentrations on pleasantness and preferences of a food. The second aim is to investigate whether fat affects the salt intensity perception and vice versa. The third aim is to investigate effects of individual fat taste sensitivity on pleasantness for fat, and if this is influenced by salt.

## Methods

### Experimental design

This study involved a complete factorial design in which participants tasted and rated a tomato soup with 4 different fat concentrations (canola oil, hereafter referred to as “fat”) and 5 different salt concentrations (NaCl, hereafter referred to as “salt”), thus 20 in total over a total of 4 sessions. The 20 samples were randomized between participants, 10 samples were tasted per session to prevent sensory fatigue. Participants came 4 times to rate the tomato soup samples, 2 times to rate the samples hedonically, and 2 times to rate the samples on intensity. After the hedonic sessions, the most preferred fat or salt concentration was assessed by a ranking test, based on liking. After the intensity sessions, discrimination ability was assessed by a ranking task in which participants rank according to intensity of saltiness or fattiness. In addition, fat taste sensitivity was established in duplicate by determination of the threshold of oleic acid (C18:1), which was assessed in 2 separate sessions.

### Subjects

Subjects were recruited at Deakin University, Burwood, Victoria, Australia. Fifty participants enrolled in the study, one participant dropped out after the first session because he did not like the test food. Of the remaining 49 participants (12 males), 2 male participants did not finish the study due to overseas travelling. Participants were aged between 18 and 54 years (27±8 years, mean ± SD). The BMI range was between 16.7 and 34.2kg/m^2^ (23.2±3.8kg/m^2^, mean ± SD). Exclusion criteria were: smoking; gained, or lost > 5kg weight during the last year; lack of appetite; and difficulties with eating or swallowing. Participants provided informed written consent prior to participation. This study was conducted according to the guidelines laid down in the Declaration of Helsinki and all procedures involving human subjects were approved by the Deakin University Human Research Ethics Committee. Pleasantness and intensity procedures were conducted in computerized, partitioned sensory booths using Compusense Five Software Version 5.2 (Compusense Inc.). This study was registered (ACTRN12614000955617) at the Australian New Zealand Clinical Trials Registry (ANZCTR).

### Test foods

A tomato soup was used as the test product in this study, prepared from 50% tomato passata (Remano, Aldi) and 50% water. Canola oil (Homebrand Coles), was used to manipulate the fat concentrations: 0, 5%, 10%, and 20% (w/w). Sodium Chloride (NaCl, Saxa salt) was added to manipulate the salt concentrations: 0.04% (no added salt) 0.25%, 0.5%, 1.0%, and 2.0% (w/w). Samples were homogenized for 1min per 100g (Silverson L4RT homogenizer). These 4 different fat concentrations and 5 different salt concentrations were used for hedonic and intensity ratings. One additional fat concentration (15%), and 2 additional salt concentrations (0.35% and 0.7%) were prepared for the ranking tests (Table 1).

**Table 1.** Overview of ranking tasks after taste sessions

Taste session	Ranking task	Used fat for ranking (%)	Used salt for ranking (%)
Hedonic 1	Preferred fat concentration	5, 10, 15, 20	0.04 and 1
Hedonic 2	Preferred salt concentration	0 and 20	0.25, 0.35, 0.5, 0.7, 1.0
Intensity 1	Intensity fat	5, 10, 15, 20	0.04 and 1
Intensity 2	Intensity salt	0 and 20	0.25, 0.35, 0.5, 0.7, 1.0

Fat taste sensitivity was assessed by determining the detection threshold for C18:1 in a nonfat milk base (Haryono et al. 2014). For test sample preparation, C18:1 was mixed at varying concentrations (0.02, 0.06, 1, 1.4, 2, 2.8, 3.8, 5, 6.4, 8, 9.8, 12, and 20mM) with long-life skim milk (99.9% fat free, Devondale). Textual cues were minimized with an addition of 5% (w/v) gum acacia (Deltagen) and liquid paraffin (Merck). To prevent oxidation, samples were mixed with 0.01% (w/v) EDTA (Merck). Samples were homogenized for 30s per 100mL solution (Silverson L4RT homogenizer). Control samples were prepared in the same manner, but without the addition of C18:1.

### Procedure of hedonic and intensity ratings

Participants were instructed to refrain from drinking (except water) and eating at least 1h before the start of the session. Participants started to taste and rate 10 samples differing in salt and fat concentrations without nose clips, and rinsed their mouth with water in between samples. In the hedonic sessions participants rated pleasantness, desire-to-eat and just-about-right saltiness on a 100 unit visual analogue scale (VAS). Pleasantness was rated on a scale ranging from “very unpleasant” (0) to “very pleasant” (100). Desire-to-eat was rated on a scale ranging from, “not at all” (0), to “very much” (100). For just-about-right saltiness intensity, “not nearly salty enough” was at the left anchor (−50), “just right” in the middle (0), and “much too salty” (50) at the right anchor. In the intensity sessions, participants rated both the intensity of fattiness and saltiness on a labelled magnitude scale (LMS).

### Procedure of ranking tasks: The most preferred concentration and the discrimination ability

After tasting and rating 10 samples, participants were instructed to rank either fat or salt in an order of liking or intensity (Table 1). Five salt concentrations were used, but only 4 fat concentrations were used, as it was expected that it was more difficult to discriminate between fat concentrations. Participants ranked the 4 fat concentrations once without added salt (0.04%), and once with salt (1.0%). Similarly, they ranked the 5 salt concentrations without fat (0%) and with fat (20%) (Table 1). In the hedonic sessions, participants were instructed to rank the samples from left to right from the least preferred to the most preferred. In the intensity sessions, participants were instructed to rank the samples from left to right from the least to the most intense perceived saltiness or fattiness.

To calculate the score of the discriminatory ability, the order of the concentrations were put in a formula: Discrimination ability for salt = (−2 × c1) − (1 × c2) + (0 × c3) + (1 × c4) + (2 × c5). Where c1–c5 were the values of the concentrations in the order of the subject from low to high. The values of the concentrations were −2 for the lowest concentration (c1), −1 for the second lowest (c2), 0 for the middle (c3), 1 for the second highest concentration (c4), and 2 for the highest concentration (c5). In this way, the complete correct order means a score of 10 and the complete wrong means a score of −10 (De Graaf and Zandstra 1999). The discrimination ability for fat was calculated in similar way: (−2 × c1) − (1 × c2) + (1 × c3) + (2 × c4).

### Fat taste sensitivity

The threshold for C18:1 was assessed by an ascending method of the 3-alternative forced choice test, in duplicate at 2 different sessions (Haryono et al. 2014). Participants were instructed to refrain from drinking (except water) and eating at least 1h before the start of each session. To prevent confounding from nonoral sensory inputs, participants wore nose clips and milk samples were presented under red light conditions. The detection threshold was defined as the concentration that was correctly identified as the odd sample 3 consecutive times. The arithmetic mean detection threshold was calculated from the 2 sessions. Six participants showed a detection threshold that differed more than 3 concentrations measured at 2 different sessions. These participants were invited for a third session, the 2 closest measured detection thresholds were averaged and the outlying measurement was omitted.

### Statistical analyses

Statistical analyses were performed using SAS version 9.3 (SAS Institute, Inc.). Data on hedonic and intensity ratings are presented as mean ± SD. Effects of salt and fat concentration and their interaction on hedonic and intensity ratings were tested in a generalized linear model (PROC GLM) that included participant as repeated factor. Tukey–Kramer was used for post hoc comparisons. A quadratic response surface by least-squares regression (PROC RSREG) was used to approximate the maximal hedonic response and the corresponding salt and fat concentrations at this maximum.

The frequency distribution of the most preferred fat and salt concentrations (hedonic ranking), are presented in a histogram. Equality for frequency distributions was tested with chi-square tests. The discrimination ability for fat and salt are expressed as scores based on the intensity ranking task. The scores are presented as medians with the interquartile range (IQR 25th–75th percentile) as index of variance. Medians were compared by using the Wilcoxon Signed-Ranks Test. Participants were divided in 3 groups based on fat taste sensitivity (threshold C1:18). Medians between groups were compared by Wilcoxon Signed-Ranks Test. Spearman’s Rho was calculated for correlations between various outcome measurements.

## Results

### Pleasantness of fat and salt concentrations

**Figure 1.**
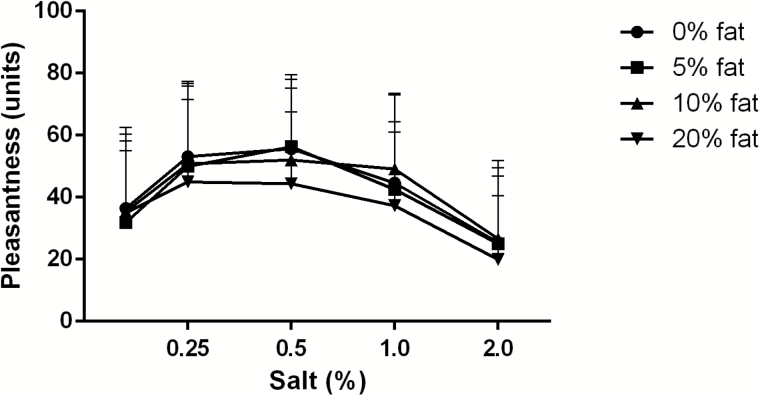
Mean + SD values of pleasantness ratings as a function of salt and fat concentrations (*n* = 47).

There was a main effect of salt [*F*(4, 892) = 47, *P* < 0.001] and a main effect of fat [*F*(3, 892) = 4.4, *P* = 0.004] on pleasantness (Figure 1), but no interaction (*P* = 0.79). Post hoc comparisons for salt (of all fat concentrations together) showed that 2% salt was least pleasant (different from other salt concentrations, all *P*-values < 0.001), followed by 0.04% salt (different from other salt concentrations, all *P*-values < 0.01), followed by 1% salt (different from other salt concentrations, all *P*-values < 0.05), whereas 0.5% salt and 0.25% salt were the most pleasant and did not differ (*P* = 0.84). Post hoc comparisons for fat (of all salt concentrations together) showed that concentrations of 0%, 5%, and 10% fat did not differ (all *P*-values > 0.78), but significantly lower pleasantness for 20% fat compared with 0% and 10% of fat (both *P*-values < 0.05). Similar effects for salt and fat were found on ratings of desire-to-eat (data not shown).

**Figure 2.**
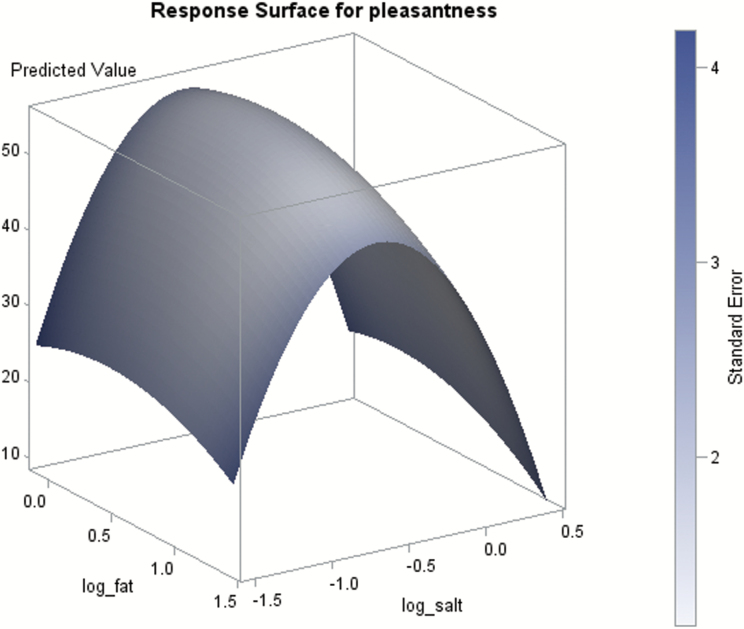
Response surface curve mapping of the maximal pleasantness as a function of salt and fat concentrations in the test food. The quadratic response surface model (*P* < 0.001) identified an optimum with the following coordinates: Pleasantness = 56mm, salt = 0.24% (salt_log = −0.61), fat = 2.2% (fat_log = 0.35) (*n* = 47).

Figure 2 shows the quadratic response surface curve mapping of pleasantness as a function of salt and fat. The model clearly shows that pleasantness was more affected by the salt concentration than by the fat concentration in the test food.

### Preferred fat concentration, with and without salt

**Figure 3.**
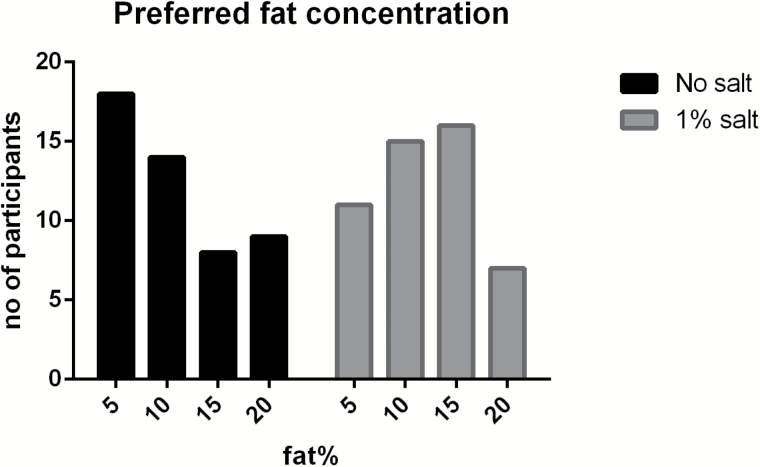
Frequency distribution of preferred fat concentration without salt (black) and with 1% salt (gray) (*n* = 49).

Figure 3 shows the frequency distribution preferred fat concentration without added salt and with added salt (1%). Frequencies of preferred fat without salt (*P* = 0.15) and with salt (*P* = 0.25) were equally distributed according to chi-square tests.

### Preferred salt concentration, with and without fat

**Figure 4.**
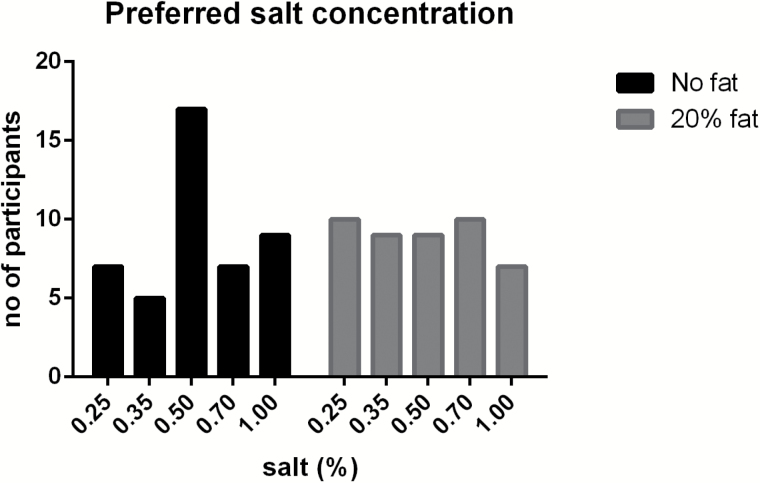
Distribution of preferred salt concentration without fat (black) and with 20% fat (gray) (*n* = 45).

Figure 4 shows that the preferred salt concentration without fat is 0.5%, whereas with fat, the preference for salt was spread out over different salt concentrations. Chi-square tests for equal distributions showed that salt preference without fat was not equally distributed (*P* = 0.04), whereas with fat, salt preference was equally distributed over the salt concentrations (*P* = 0.96).

### Intensity of fat and salt concentrations

**Figure 5.**
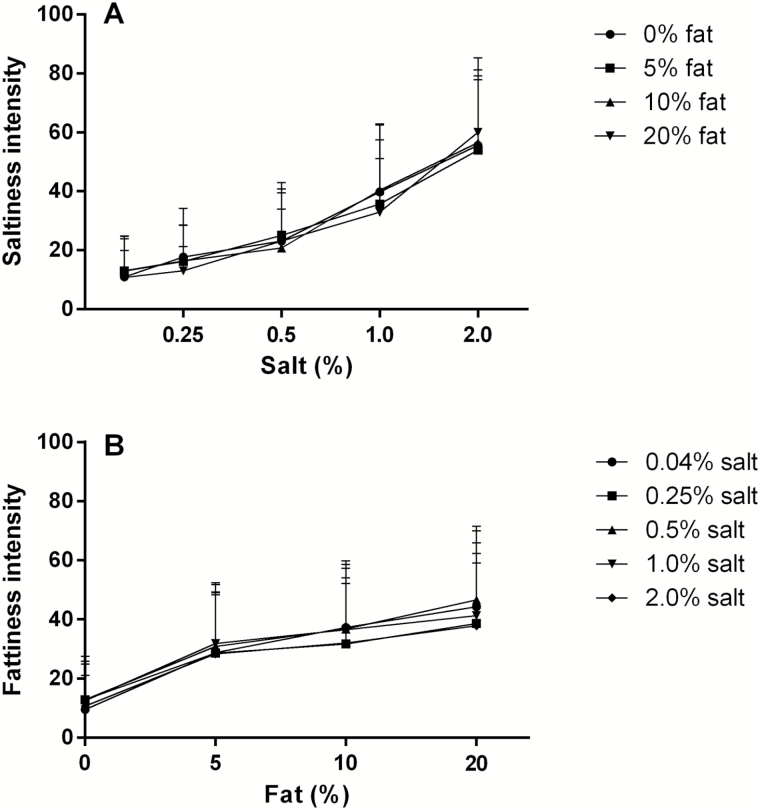
Mean + SD saltiness intensity ratings of increasing salt concentrations (**A**) and fattiness intensity ratings of increasing fat concentrations (**B**) (*n* = 48).

The intensity of saltiness is affected by the salt concentration [*F*(4, 882) = 273, *P* < 0.001], and there was no interaction with fat (*P* = 0.29) (Figure 5A). The intensity of fattiness is affected by the fat concentration [*F*(3, 882) = 147, *P* < 0.001], and there was no interaction with salt (*P* = 0.77) (Figure 5B).

### Discrimination ability of fat and salt concentrations

The median score for the ability to discriminate between 5%, 10%, 15%, and 20% of fat was 6 (IQR 1–9) without salt and 3 (IQR 1–9) with 1% salt in the test food (*P* = 0.87). The median score for the discrimination ability for salt was 9 (9–10) without fat and 9 (8–10) with 20% fat in the test food (*P* = 0.94). In general, the score of the discrimination ability was much higher for salt (with and without fat) than for fat (with and without added salt) (*P* < 0.001).

The discrimination ability for fat without added salt tend to be negatively correlated with the preferred fat concentration without salt (Rho = −0.27, *P* = 0.065), indicating that participants who prefer higher fat concentrations were less able to discriminate between different fat concentrations. This effect was not observed in salt.

The discrimination ability for salt was positively correlated with the slope for the ratings of “just-about-right” saltiness (Rho = 0.36, *P* = 0.02 for discrimination ability without fat, Rho = 0.24, *P* = 0.10 for discrimination ability with fat). A steep slope means that participants have high hedonic sensitivity to saltiness, whereas a weaker slope means that participants have a low hedonic sensitivity to saltiness (all salt concentrations are closer to the “just right” saltiness). This means that participants who discriminate better between different salt concentrations do also show more hedonic differences to salt.

### Effects of fat taste sensitivity on fat and salt preference and perception

**Figure 6.**
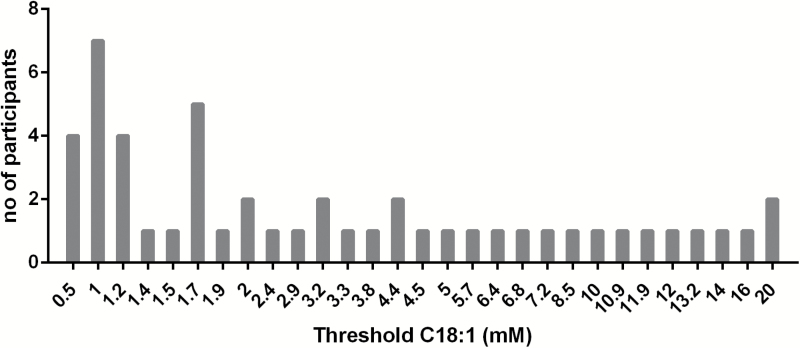
Frequency distribution of measured detection thresholds of C18:1 of all participants (*n* = 49).

Table 2 shows differences in preferred fat and salt concentrations, and discrimination ability. Figure 6 shows the frequency distribution of the detection threshold of C18:1. The median threshold of C18:1 was 2.0mM (IQR 1.2–6.6), participants were split into 3 groups with equal number of participants based on their sensitivity. Participants with higher fat taste sensitivity (i.e., lower detection thresholds of C18:1) preferred lower fat concentrations, but only without added salt. This is most obvious when looking at the interquartile range and the frequency distribution (data not shown); only one subject selected the 20% fat as most pleasant in the most sensitive group, whereas 6 participants selected the 20% fat concentration in the least sensitive group (without added salt). There were no other differences observed in preferences for fat and salt or in discrimination ability.

**Table 2.** Detection threshold C18:1, preferences, and discrimination ability between groups classified on fat taste sensitivity, data presented as medians and IQR

	Fat taste sensitivity group (range detection threshold C18:1mM)			*P*
Group 1 (*n* = 17) (threshold 0.5–1.5)	Group 2 (*n* = 16) (threshold 1.7–4.4)	Group 3 (*n* = 16) (threshold 5–20)
Detection threshold C18:1 (mM)	1 (0.8–1.2)	2.2 (1.7–3.3)	10.5 (6.5–13.8)	**<0.001**
Preferred fat concentration (%) (no salt)	10 (5–10)	12.5 (5–15)	12.5 (5–20)	**0.008**
Preferred fat concentration (%) (1% salt)	15 (10–17.5)	10 (5–15)	15 (6.25–15)	0.30
Discrimination ability fat (score) (no salt)	9 (3–9.5)	6 (-3–9)	8 (2–10)	0.53
Discrimination ability fat (score) (1% salt)	6 (3–9)	3 (1–9)	3 (1–9)	0.58
Preferred salt concentration (%) (no fat)	0.5 (0.5–1)	0.5 (0.27–0.7)	0.5 (0.35–0.7)	0.88
Preferred salt concentration (%) (20% fat)	0.5 (0.35–0.7)	0.5 (0.25–0.7)	0.43 (0.35–0.7)	0.97
Discrimination ability salt (score) (no fat)	9 (8–10)	9 (9–10)	9 (8–10)	0.99
Discrimination ability salt (score) (20% fat)	9 (9–10)	9 (9–10)	9 (3–10)	0.58

Bold *P*-values indicate significant difference.

## Discussion

The present study shows effects of both fat and salt on pleasantness and taste perception within a food matrix. Salt concentration (ranged from 0.04% to 2%) had more impact on pleasantness than fat concentration (0–20%). There was no interaction of fat and salt on pleasantness or taste perception.

The primary aim was to investigate contributions and interactions of salt and fat on pleasantness and preferences of a food. The present study shows that salt has a major impact on pleasantness, as also found in other studies (Booth et al. 1983; Shepherd et al. 1984; Bolhuis et al. 2010, 2012). The response surface curve mapping (Figure 2) and the pleasantness curve (Figure 1) show that pleasantness was very closely related to salt concentration, and less related to fat concentration. The most pleasant concentration of salt was around 0.25–0.5%, according to the pleasantness curves (Figure 1), response surface mapping (Figure 2), and most preferred saltiness (Figure 4). There was no clear hedonic breakpoint for fat, we expected that low fat concentrations would slightly increase pleasantness. However, fat concentrations of 0%, 5%, and 10% were not significantly different in pleasantness, whereas 20% of fat was less pleasant. Abdallah et al. (1998) used a similar design and investigated different sugar and fat concentrations in a solid food matrix. In accordance to our study, pleasantness was less determined by fat and more by sugar concentration.

In line with poor hedonic differences in fat concentrations, participants also poorly discriminated the intensity between fat concentrations. Participants discriminated much better between different salt concentrations, and also show more hedonic differences between salt concentrations. In accordance, other studies showed that participants had poor perception of different fat concentrations (Abdallah et al. 1998; Drewnowski and Schwartz 1990), but preferred the moderate fat (Abdallah et al. 1998) or highest fat sample (Drewnowski and Schwartz 1990). It has been suggested that preference for high-fat stimuli is not based on a conscious perception of the fat content (Drewnowski and Schwartz 1990; Mela 1990). This is reinforced by a recent study demonstrating there was no discrimination in the primary taste cortex for high vs. low fat, but high-fat induces reward responses in the brain (Tzieropoulos et al. 2013). Given that humans poorly discriminate between different fat concentrations, but fat is related to activation of reward areas, suggests that implicit [i.e., unconscious, spontaneous reaction (Berridge 2004)] measurements (e.g., working tasks, food intake) maybe more useful for measuring preferences for fat than line scales.

The second aim of the study was to investigate whether fat affects the salt intensity perception and vice versa. No interactions were found for fat and salt on saltiness or fattiness intensity ratings. In line with this, the discrimination ability for salt was not affected by increasing fat concentration and vice versa. In accordance, others did not find salt (0–0.6%) and fat (0.5–36%) interactions in a dairy liquid product on hedonics (Warwick and Schiffman 1990), or found salt and fat (9% and 17%) interactions on saltiness intensity in oil-in-water emulsions (Metcalf and Vickers 2002). However, Suzuki et al. (2014) investigated effects on saltiness intensity for 0%, 10%, 20%, and 40% oil-in-water emulsions as function of salt concentration and reported differences in saltiness intensity diminished with increasing oil concentrations, especially at 40% oil. The present study did not use higher concentrations than 20% oil. Although we did not find a significant interaction between salt and fat, Figure 4 suggests a diminished hedonic sensitivity for salt in the 20% fat containing soup. In 20% fat, the preference for saltiness is spread out into different concentrations, whereas a clear preference is observed for 0.5% salt in the 0% fat soup. Whether 20% or higher concentrations of fat affects salt perception and pleasantness needs to be studied further, preferably in semi-solid or solid foods because higher levels of fat are more common and seemed to be better liked in solid than in liquid foods (Drewnowski et al. 1989; Drewnowski and Schwartz 1990).

Surprisingly, the present study does not show an increase in pleasantness when fat is added to the food. We expected to find an increase in pleasantness in the 5% and 10% fat soups, but pleasantness did not differ between the soups with 0%, 5%, and 10% fat. Studies generally report a positive relationship between liking and fat content, although there are some mixed results (see for review, Cox et al. 2015). Different test foods may explain the variation in results. The test food in the present study was a tomato drink that was associated with a cold/room temperature soup. However, participants in the present study might be unfamiliar with fat in the tomato soup, which may explain that we did not find an increase in pleasantness. Another explanation could be the choice of canola oil, which is possibly less palatable than for example cream. Possibly, using cream instead of canola oil may have led to stronger effects on pleasantness, but such a major impact on pleasantness as salt would be unlikely. Canola oil was used as it has the highest percentage of mono-unsaturated fatty acids compared with other commonly used oils, which was aimed to be linked to fat taste sensitivity measured using oleic acid (C18:1). Although no gravity or separation of the oil and water phases have been observed and the fact that all samples were freshly prepared and stirred thoroughly just before assessment, a possible negative effect of emulsion stability on hedonics cannot be excluded (Granato et al. 2010).

The average and low pleasantness scores of the tomato soup in general may not be representative to palatable salty and fatty foods that easily lead to overconsumption. Nevertheless, the low score of the tomato soup without fat and salt was ideal to observe increases in pleasantness due to additions of salt and fat. A main outcome of this study was no interaction of salt and fat on pleasantness and taste perception. Also others did not find salt and fat interactions on pleasantness (Warwick and Schiffman 1990) and on saltiness intensity in liquid foods (Metcalf and Vickers 2002), therefore, this is considered to be wider applicable to other savory liquid foods. Another main outcome is the relative importance of salt on pleasantness compared to fat. This reflects the challenge of the food industry to reduce salt while maintaining palatability (Dotsch et al. 2009), whereas low-fat or even 0% fat foods seem to be more common and widely accepted (e.g., like 0% fat yoghurts and custards).

The third aim of the study was to investigate how individual fat taste sensitivity affects preferences for fat, and whether this is influenced by salt. The results show that sensitive participants (i.e., low threshold for C18:1), preferred lower fat concentrations compared with less sensitive participants. This effect was only observed in the food without salt, suggesting that salt masks these fat preferences. We did not observe a relationship of fat taste sensitivity on discrimination ability for fat concentrations, in contrast to a previous study (Stewart and Keast 2012a). The study of Stewart and Keast (2012a) used nose clips to exclude odor attributes and corrected for textural differences. The present study did not use those corrections when tasting the tomato soup, because hedonics of fat and salt combinations was the primary aim. Fat perception is a combination of textural, odor, and taste attributes. The specific role of taste on fat perception in suprathreshold fat concentrations is not clear (Keast and Costanzo 2015). No odor blocking or texture masking was used in this study, which could explain the lack of relationship between taste sensitivity and discrimination ability.

## Conclusion

This study shows that salt is more closely related to pleasantness than fat in a savory liquid food. The passive role of fat on pleasantness found in this study and others indicates that fat content may be reduced while maintaining palatability, however, this will depend on the food matrix as well. The strong effect of salt on pleasantness may indicate that salt is major driver of food intake of savory fatty foods, and reflects the challenge to reduce salt while maintaining palatability (Dotsch et al. 2009). Relationships between salt taste responses and overconsumption or obesity have been suggested (Méjean et al. 2014; Cox et al. 2015), however, received little attention in literature compared with sweet or fat taste responses. Future research is needed to investigate whether high fat concentrations (20% in the present study) decrease hedonic sensitivity to salt.

## Funding

The study was supported by National Health and Medical Research Council (1043780) and the School of Exercise and Nutrition Sciences of Deakin University, Vic, Australia.
